# Rethinking gastroenteropancreatic neuroendocrine neoplasms: A perspective on cellular origins, molecular mechanisms, and combination therapies

**DOI:** 10.1515/jtim-2026-0035

**Published:** 2026-04-18

**Authors:** Huiying Shi, Shizhao Xu, Rong Lin

**Affiliations:** Department of Gastroenterology, Union Hospital, Tongji Medical College, Huazhong University of Science and Technology, Wuhan, Hubei Province, China

## Introduction

Neuroendocrine neoplasms (NENs) are a highly heterogeneous group of malignancies originating from neuroendocrine cells and peptidergic neurons throughout the body, and their incidence has increased remarkably over recent decades. The lack of a unified biological framework impedes a precise understanding of NEN biology, undermines reliable prognostic assessment, and complicates treatment development.

Recent advances in multi-omics technologies are catalyzing a transformative paradigm shift in NEN research, prompting a re-examination of cellular origins and molecular classifications. Combination therapy strategies are also being actively explored. As gastroenteropancreatic neuroendocrine neoplasms (GEP-NENs) account for 55%–70% of all NENs, we focus on GEP-NENs to illustrate these advances.

## Cellular origin

The cellular origins of GEP-NENs are complex and incompletely understood. While pancreatic neuroendocrine tumors (PanNETs) were traditionally thought to arise from endocrine cells, recent evidence supports a more diverse pathogenesis. Saghafinia *et al*. identified an invasive metastasis-like primary (MLP) subtype, possibly arising from islet tumor dedifferentiation with β-cells reverting to a progenitor state.^[1]^ The concept of cellular plasticity is reflected in the 2022 WHO classification of non-functional pancreatic NETs (NF-PanNETs), which defines α-like, β-like, and intermediate subtypes based on epigenetic and transcriptomic profiles. α-like tumors arise from α-cells after MEN1 loss and may dedifferentiate into intermediate tumors *via* alterations like *DAXX/ATRX* inactivation and chromosomal instability, whereas β-like tumors originate from β-cells through distinct genetic events. Meanwhile, Moser *et al*. identified a D subtype for PanNETs, suggesting a possible origin from intestinal-type endocrine cells near the ampulla and duodenum.^[2]^

In contrast, neuroendocrine carcinomas (NECs) appear to have distinct origins. Phylo-epigenetic analyses suggest that PanNECs arise from pancreatic acinar cells, as they cluster more closely with exocrine cells and exhibit an acinar-like cell signature. ^[3]^ In small cell lung cancer, pulmonary basal cells with *MYC* amplification, *PTEN* loss, and *ASCL1* suppression—can transdifferentiate into malignant neuroendocrine phenotypes.^[4]^ Similarly, in GEP-NECs, introduction of *TP53* and *RB1* mutations plus overexpression of key transcription factors in Lgr5^+^ colon organoids can drive transformation into NEC.^[5]^

Therefore, understanding GEP-NENs origins hinges on shifting from a static “cell-of-origin” perspective to a dynamic analysis of cellular plasticity. This plasticity-based paradigm not only unifies the continuum from well-differentiated NETs to poorly differentiated NECs, but also informs novel therapies targeting core tumorigenic mechanisms like dedifferentiation and transdifferentiation.

## Molecular mechanisms

Building on insights into cellular origin and plasticity, the molecular mechanisms driving GEP-NEN pathogenesis display distinct, yet still incompletely defined, patterns. NENs are hereditary or sporadic. In hereditary GEP-NENs, *MEN 1*, *VHL*, and *RET* mutations are established pathogenic drivers.^[6]^ Sporadic GEP-NENs instead show site- and differentiation-specific mutation profiles. Most PanNETs harbor *MEN1* and/or *DAXX/ATRX* mutations. *MEN1* mutations cause transcriptional dysregulation, mTOR activation, and defective DNA repair, whereas *DAXX/ATRX* mutations promote alternative lengthening of telomeres and chromosomal instability. Conversely, most PanNECs harbor *TP53* and *RB1* alterations linked to cell-cycle dysregulation, while non-pancreatic NECs exhibit *TP53* mutations, *RB1* alterations, and/or *CCNE1/MYC* amplification, along with inactivation of Notch family genes.

Beyond genetic mutations, epigenetic alterations are key contributors to GEP-NENs pathogenesis. DNA methylation and histone modifications can reshape gene expression without changing DNA sequence. For example, promoter hypermethylation of tumor suppressor genes (*e.g*., *RASSF1, HIC1, APC, CDKN2A*, *MGMT,* and *TIMP3*) is common in PanNETs and silences their transcription.^[7]^ Epigenetic landscapes also differ across GEP-NEN subtypes: PanNET G3 shows hypomethylation of α-cell markers (*e.g*., *IRX2, TTR, GLS*), whereas PanNEC shows hypo- or hypermethylation of multiple exocrine cell-related gene promoters.^[3]^ However, whether these alterations are initiating events requires further investigation.

## Molecular subtyping

According to the 2022 WHO classification, NETs are graded G1-G3 by mitotic count and Ki-67 index, but this scheme has prognostic and therapeutic limits. For example, some low-grade tumors, such as rectal NET G2, still show appreciable metastatic risk.^[8]^ Therefore, the current grading system does not fully capture biological behavior, and research is increasingly shifting toward molecular subtyping. Recent work has proposed classifying PanNETs using transcriptomic and proteogenomic data. Transcriptomically, PanNETs can be classified into four subtypes: MLP-1, MLP-2, insulinoma-like, and intermediate.^[9]^ The MLP-1 subtype displays an immunologically “hot” but suppressive tumor microenvironment, with dense immune infiltration and upregulation of high expression of immune checkpoints such as PD-L1.^[9]^ Proteogenomic analyses refine PanNET stratification by integrating genomic, proteomic, and functional pathway data. In the study by Ji *et al*., NF-PanNETs were classified into four distinct molecular subtypes (C1–C4).^[10]^ C1 shows an immunosuppressive microenvironment and the poorest outcome; C2 has an α-cell-like phenotype with frequent *MEN1/DAXX/ATRX* mutations and an immune-cold profile; C3 displays a β-cell-like signature with proliferative pathway activation, also immune-cold; and C4 is defined by VHL mutations, upregulated hypoxia/glycolysis/myogenesis pathways, and a mixed immune phenotype.

These molecular subtypes, defined by multi^‑^omics technologies, underscore NEN heterogeneity and help guide individualized clinical treatment. Immunologically “hot” but suppressive subtypes with poor prognosis, such as the MLP-1 transcriptomic subtype and proteogenomic C1 subtype, may benefit from immunotherapy, whereas subtypes with specific pathway activation provide targets for tailored therapy.

## Treatment

Currently, treatment for GEP-NENs generally follows a stepwise, single-agent strategy, using somatostatin analogs (SSAs; *e.g*., octreotide, lanreotide), targeted agents (*e.g*., everolimus, sunitinib), cytotoxic chemotherapy (*e.g*., temozolomide-based regimens, platinum–etoposide for NECs), and peptide receptor radionuclide therapy (PRRT). Although immune checkpoint inhibitors have shown striking efficacy in colorectal and gastric cancers, their monotherapy objective response rate (ORR) in GEP-NENs is low. Nevertheless, growing numbers of trials are testing combination regimens, including checkpoint blockade, in advanced or aggressive GEP-NENs. Here, we systematically review the key findings of these studies, summarized in Table 1.

**Table 1:** Summary of clinical trials of therapeutic combinations for GEP-NENs

Trial names	Trial interventions	Patients	Primary endpoints
STARTER-NET Phase III (jRCT1031200023)	Everolimus+Lanreotide *vs*. Everolimus	G1 or G2, nonfunctioning GEP-NETs with poor prognostic factors (Ki-67 labeling index (LI) 5%-20% or diffuse liver metastases)	PFS: 29.7 mo *vs*. 11.5 mo
RADIANT-2 Phase III (NCT00412061)	Everolimus+Octreotide LAR *vs*. Octreotide LAR	Low- or intermediate-grade, advanced, colonic or rectal NETs	median PFS: 29.9 mo *vs*. 6.6 mo
SONNET Phase II (NCT02231762)	Lanreotide+Temozolomide	Progressive advanced/metastatic G1/G2 GEP-NET or of unknown primary	6-month DCR: 73.5%;
AXINET Phase II (NCT01744249)	Octreotide LAR+ Axitinib *vs*. Octreotide LAR	Advanced G1/G2 extra-pancreatic NETs	ORR: 13.2% *vs*. 3.2%; median PFS: 16.6 mo *vs*. 9.9 mo
CALGB 80701 Phase II (NCT02795858)	Everolimus+Bevacizumab *vs*. Everolimus	Locally unresectable or metastatic, well or moderately differentiated PanNETs	CR/PR: 31% *vs*. 12%
NETTER-2 Phase III (NCT03972488)	177Lu-Dotatate+octreotide 30 mg LAR *vs*. high-dose octreotide 60 mg LAR	Patients with newly diagnosed higher grade 2 (Ki67 ≥ 10% and ≤ 20%) and grade 3 (Ki67 > 20% and ≤ 55%), somatostatin receptor-positive (in all target lesions), advanced GEP-NETs	PFS: 22.8 mo *vs*. 8.5 mo; ORR: 43.0% *vs*. 9.3%
Phase II (NCT04169672)	Surufatinib+Toripalimab	Advanced NETs or NECs/MiNENs who had failed or were intolerable of standard treatment	ORR: 21.1% and 23.8%; PFS: 9.6 and 4.1 mo; OS: 27.3 and 10.9 mo
Phase II (NCT03475953)	Regorafenib+Avelumab	Advanced G2-G3 well-differentiated GEP-NETs or G3 GEP-NECs after progression on prior therapies	6-month ORR: 18%; median PFS: 5.5 mo
NICE-NEC Phase II (NCT03980925)	Nivolumab+Platinum-Etoposide	Chemotherapy-naive, unresectable advanced or metastatic G3 NENs of gastroenteropancreatic or unknown origin	ORR: 56.8%; median PFS: 5.7 mo; median OS: 13.9 mo.

GEP-NENs: gastroenteropancreatic neuroendocrine neoplasms; NET: neuroendocrine tumor; NEC: neuroendocrine carcinoma; MiNEN: mixed neuroendocrine–non-neuroendocrine neoplasm; LAR: long-acting repeatable; PanNET: pancreatic neuroendocrine tumor; ORR: objective response rate; DCR: disease control rate; PFS: progression-free survival; OS: overall survival; CR: complete response; PR: partial response; mo: months.

In the phase III STARTER-NET trial, first-line everolimus plus lanreotide prolonged median progression-free survival (PFS) to 29.7 *vs*. 11.5 months with everolimus alone in unresectable or recurrent GEP-NETs. Similarly, the phase III NETTER-2 trial demonstrated that first-line [^177^Lu] Lu-DOTA-TATE plus octreotide LAR prolonged median PFS to 22.8 *vs*. 8.5 months with high-dose octreotide LAR alone in patients with SSTR-positive, G2–G3 advanced GEP-NETs. Other phase II/III trials have also reported benefits for combinations of SSAs with chemotherapy, molecular targeted drugs, or anti-angiogenic drugs.

Combination immunotherapy is a key focus in NENs. In a Chinese phase II trial (NCT04169672), surufatinib plus toripalimab achieved ORRs of 21.1% in pretreated advanced NET and 23.8% in NEC/ mixed neuroendocrine non-neuroendocrine neoplasms (MiNENs) cohorts. The higher ORR observed in the NEC/MiNEN cohort supports the hypothesis that these more aggressive histologies are associated with a greater mutational burden and increased neoantigen presentation. Additionally, the phase II NICE-NEC trial evaluated nivolumab plus platinum-etoposide in treatment-naive advanced grade 3 GEP-NENs, reporting an ORR of 56.8%, median PFS of 5.7 months, and median OS of 13.9 months. This activity likely reflects synergy with chemotherapy, which provides direct cytotoxicity and enhances immunotherapy by promoting immunogenic cell death, reducing regulatory T-cell activity, and inducing PD-L1 expression.

While combination therapy is a promising treatment strategy, the current evidence remains insufficient. Although some regimens (*e.g*., everolimus plus SSAs, PRRT plus SSAs) are supported by phase III trials, many others are derived from phase II studies. These trials lack parallel control groups and have limited sample sizes, which limits direct comparisons with standard treatments. Therefore, large-scale, prospective validation is needed to confirm efficacy and guide clinical application.

Therapeutic innovation for GEP-NENs includes novel targets like HDAC inhibitors. The class I inhibitor entinostat showed efficacy in a phase II trial (NCT03211988), prolonging stability and reducing tumor growth by 32%–83% in refractory NETs. Nonetheless, these early results are constrained by limited sample sizes and follow-up, underscoring the demand for continued research.

## Conclusions

In summary, multi-omics research has profoundly advanced our understanding of GEP-NENs, moving beyond morphology to reveal dynamic cellular origins, complex molecular mechanisms, and distinct biological subtypes. To translate these insights into practice, future work should focus on validating molecular classifications, confirming combination therapies in large, prospective phase III trials, and identifying new therapeutic targets. Together, these efforts may usher in a new era of precision medicine for GEP-NENs and improve patient outcomes.
